# The Games We Play: Prosocial Choices Under Time Pressure Reflect Context-Sensitive Information Priorities

**DOI:** 10.1177/09567976221094782

**Published:** 2022-08-22

**Authors:** Yi Yang Teoh, Cendri A. Hutcherson

**Affiliations:** 1 Department of Psychology, University of Toronto; 2 Department of Marketing, Rotman School of Management, University of Toronto

**Keywords:** attention, decision-making, cooperation, time pressure, altruism, prosocial behavior, prosociality, social cognition, morality, mouse tracking, open data, open materials, preregistered

## Abstract

Time pressure is a powerful experimental manipulation frequently used to arbitrate between competing dual-process models of prosocial decision-making, which typically assume that automatic responses yield to deliberation over time. However, the use of time pressure has led to conflicting conclusions about the psychological dynamics of prosociality. Here, we proposed that flexible, context-sensitive information search, rather than automatic responses, underlies these divergent effects of time pressure on prosociality. We demonstrated in two preregistered studies (*N* = 304 adults from the United States and Canada; Prolific Academic) that different prosocial contexts (i.e., pure altruism vs. cooperation) have distinct effects on information search, driving people to prioritize information differently, particularly under time pressure. Furthermore, these information priorities subsequently influence prosocial choices, accounting for the different effects of time pressure in altruistic and cooperative contexts. These findings help explain existing inconsistencies in the field by emphasizing the role of dynamic context-sensitive information search during social decision-making, particularly under time pressure.

Humans sometimes sacrifice self-interest to benefit others, but debates about why and how we act so prosocially have persisted throughout history (Henrich et al., 2005; Herbert, 1993; Persky, 1995). Recent work suggests that these prosocial behaviors result from competing dispositional social preferences that evolve over time, from more intuitive to more deliberate preferences (Chen & Krajbich, 2018; Hallsson et al., 2018; Kahneman & Egan, 2011; Moore & Loewenstein, 2004; Rand et al., 2012). Although there are several different dual-process theories of social preferences that vary in their precise characterization of the underlying nature of social intuitions (Haidt, 2001; Moore & Loewenstein, 2004; Plötner et al., 2021; Rand, 2016; Zaki & Mitchell, 2013), they all generally assume that intuitive preferences unfold quickly and automatically, whereas reflective preferences require slow, controlled deliberation. Thus, identifying whether and why peoples’ intuitions tend toward prosociality or self-interest may generate critical insight into humanity’s social nature and its evolutionary origins (Bear & Rand, 2016; Haidt, 2001).

To uncover these intuitions, researchers often apply time pressure during prosocial choice, assuming that this constrains deliberative processing. However, these methods have led to conflicting results; people sometimes become more selfish under time pressure (Capraro & Cococcioni, 2016; Krawczyk & Sylwestrzak, 2018; Teoh et al., 2020) and sometimes more prosocial (Bouwmeester et al., 2017; Rand, 2016; Rand et al., 2012). One possible explanation for such conflicting results is that individuals differ in their intuitions; some are predisposed toward prosociality and others toward selfishness (Chen & Krajbich, 2018; Cornelissen et al., 2011; Pancotto & Righi, 2021). But this does not fully explain seemingly large and systematic differences in the effects of time pressure across studies. Thus, an important set of questions remains: How and why does time pressure change prosocial behavior, and does it reveal intuitive social dispositions?

Here, we propose that inconsistencies in time pressure’s effects in part reflect the disparate contexts in which researchers have measured prosocial behavior.^1^ Specifically, some studies measure prosociality in an altruistic context, whereas others measure prosociality in a cooperative context. Notably, altruistic prosociality (encapsulated in economics and psychology by the dictator game and its variants) involves personal sacrifice for material benefits that accrue entirely to other people. In contrast, cooperative prosociality (captured by variants of the ultimatum game, public-goods game, and prisoner’s dilemma, among others) generally involves strategic sacrifices in which increasing the benefits to other individuals can also promote self-interest (Batson, 2011; Fehr & Fischbacher, 2003; Nowak & Sigmund, 1998).

Evidence suggests that these disparate prosocial contexts engage distinct processes; people generally behave more prosocially in more strategic and cooperative settings (i.e., ultimatum games) rather than altruistic contexts (i.e., dictator games; for comparisons, see Bechler et al., 2015; Charness & Gneezy, 2008; Cochard et al., 2021; Henrich et al., 2005; Larney et al., 2019). Confirming this idea, research has found that altruistic and cooperative prosociality follow distinct developmental trajectories in children (Harbaugh & Krause, 2000; Sally & Hill, 2006), recruit distinct neural structures (Yamagishi et al., 2016, 2017), and respond differently to neural stimulation of frontal brain areas (Ruff et al., 2013). Critically, many of the studies demonstrating increased selfish behavior under time pressure measured prosocial behavior within dictator games, where self-interest directly conflicts with other people’s welfare and one’s own preferences fully determine everyone’s outcomes (Krawczyk & Sylwestrzak, 2018; Teoh et al., 2020). In contrast, studies finding increased prosociality under time pressure generally employed ultimatum games, public-goods games, or prisoner’s dilemmas, where cooperation leads to maximal joint interest and may reflect strategic self-interest because one’s own outcomes structurally depend on others (Rand, 2016). Although this might suggest that automatic intuitions differ in the two contexts, no research has directly compared the effects of time pressure across these different contexts or investigated the mechanisms that may facilitate these differences.

**Statement of Relevance**

Since antiquity, people have theorized about the nature of human sociality. Debates about whether people are fundamentally selfish or prosocial continue to occupy philosophers and laypeople alike. Modern empirical research into this question often employs the use of time pressure as an arbitrator, assuming that it reveals automatic and intuitive dispositions. However, this method has yielded persistent contradictions in our understanding of prosociality. In contrast, growing research shows that people strategically adapt information-search strategies to meet resource constraints (e.g., time pressure) during decision-making. By comparing information search during purely altruistic or cooperative choices, we found that time pressure drives adults in the United States and Canada to strategically attend to context-relevant information in a manner that constrains their subsequent choices. These findings present a potential resolution to persistent discrepancies within the field of prosocial decision-making and illustrate how social choices under constraints may reflect structural features of the choice context rather than people’s fundamental social dispositions.

Here, we drew on literature showing that people allocate cognitive resources to optimize processing under constraints (Callaway et al., 2021; Lieder & Griffiths, 2020; Teoh et al., 2020) and propose an alternative mechanistic explanation for these divergences: Altruistic versus cooperative contexts might produce different strategic information-search priorities, which in turn interact with time pressure to produce different behavioral outcomes. On the basis of this logic, and given that the majority of individuals weight their own self-interest over others’ welfare, we predicted that in dictator games, where self-interest conflicts directly with others’ welfare, people should typically prioritize self-relevant information and make less prosocial choices (replication: Teoh et al., 2020). In contrast, in ultimatum games, where one’s own outcomes depend on the other person’s acceptance of an offer, people should strategically consider others’ welfare more, both in how they choose and in what information they attend to. Furthermore, we predicted that although differences in search strategies might exist even under free response conditions, time pressure should amplify the differences in information priorities across conditions because of resource-rational adjustments in attention allocation. Finally, because time pressure might force people to choose before sampling all choice-relevant information, it should magnify the biasing influence of these different search strategies on behavior. We hypothesized that this constellation of rational adaptations in information search, when taken together, might exacerbate existing differences in behavior across social contexts and parsimoniously explain previously puzzling inconsistencies in the literature.

## Open-Practices Statement

All experimental procedures and analyses for Study 1 were preregistered prior to data collection and can be found at https://osf.io/zfrhb/. All experimental procedures and analyses for Study 2 were preregistered prior to data collection and can be found at https://osf.io/zx7b8/. Comprehensive documentation of all preregistered results is indicated in the Results section as well as in the notes of Tables S1 to S3 in the Supplemental Material available online, and all post hoc analyses are explicitly labeled. All experimental materials, deidentified preprocessed data, and analysis scripts for Studies 1 and 2 can be found at https://osf.io/ftxsc/.

## Method

### Overview

**Fig. 1.**
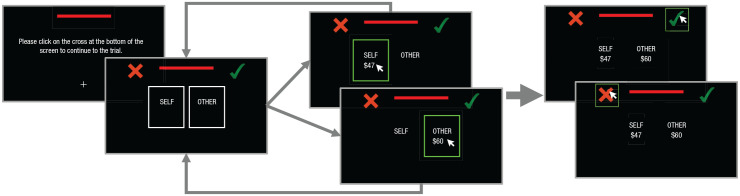
Schematic of the task in Study 1. Participants began each trial by using the mouse to click on a central location. They then revealed information by choosing to click within the “self” or “other” areas, or they made a choice by clicking on either the green check mark (accept the proposal) or the red cross (choose the default). The length of the red bar at the top of the screen signaled whether participants had 3 s or 10 s to make their choice.

To test these hypotheses, we conducted two preregistered experimental studies that manipulated time pressure and measured its influence on participants’ information-search strategies and subsequent prosocial decisions in dictator and ultimatum games. In Study 1, we recruited a random sample of participants from the United States and Canada through Prolific Academic (final *N* = 100; gender: 49 men, 50 women, one nonbinary; age: *M* = 30.3 years, *SD* = 9.90, range = 18–57 years). Each participant was randomly assigned to play either the dictator game (*n* = 50) or ultimatum game (*n* = 50) and completed 220 trials of the respective game in 10 blocks of 22 trials. Because of the novelty of paradigm, sample sizes in each of the conditions were determined on the basis of prior studies investigating effects in these games. Participants in both games encountered a series of decisions between two predetermined distributions of money for themselves and another person (see Fig. 1 for task schema). One of those distributions was always a fair split of $50 each. Participants were presented with the alternative distribution on screen on each trial and asked to accept or reject the alternative distribution versus the default. For the ultimatum game, participants were informed that their assigned partner could accept or reject their choice regarding the alternative distribution. If their partner accepted their decision, that choice would be implemented. If their partner rejected their decision, they would both forfeit the monetary outcomes and receive $0. However, participants were informed that they would not receive real-time feedback and would find out their partner’s choice on only one trial randomly selected at the end of the experiment, which would determine both their and their partner’s bonus. In contrast, dictator game participants were informed that their partners had no say over the final outcome of their decisions and their choice alone would fully determine both their and their partners’ bonus.

Importantly, to measure information search in both tasks, participants were instructed to use their mouse to click on predefined areas of the screen to sequentially reveal the outcomes for themselves and another person as they made their choice on a trial. To start the trial, participants had to click on a central cross. This served to reset and standardize the mouse position at the start of each trial. Information about payments for self and other was located in areas of interest (AOIs) hidden behind two rectangular masks highlighted by a white border and displayed on the right and left sides of the screen. These AOIs were defined as rectangles with a width of 27% of the screen width and a height of 42% of the screen height. The boundaries of the AOIs were horizontally separated from each other by a relative width of 15% and from the center by 7.5%. Both AOIs were vertically centered. The location of self and other information was consistent across all trials but counterbalanced across participants. Clicking one of the two rectangular masks revealed the respective outcome for approximately 300 ms before it disappeared. The exposure duration for each information sample was drawn from a random normal distribution of log-transformed fixation durations (milliseconds) reported for eye-tracking data from prior studies (Teoh et al., 2020; high time pressure: *M* = 5.16, *SD* = 0.73; low time pressure: *M* = 5.47, *SD* = 0.74) and exponentially transformed back into milliseconds. After exposure terminated, the mask reappeared and the participant was free to click on any further information they wished to sample. Each instance of information exposure was defined as a single information sample. Participants were free to make a choice any time throughout the duration of the trial by clicking on the green check mark to accept the proposal or red cross to reject the proposal. To manipulate time pressure, we had participants choose either within 3 s after trial onset (high time pressure) or within 10 s (low time pressure). In the low-time-pressure condition, participants could not choose within 3 s of trial onset, although they were free to sample information during this time. Participants were presented with blocks of 22 trials in the same condition, and blocks were organized such that the practice and first block consisted of low-time-pressure trials (to familiarize participants with the experimental setup), and the second block consisted of high-time-pressure trials. Subsequently, blocks were pseudorandomly interleaved such that participants completed no more than two blocks in a row of the same condition and completed five blocks (i.e., 110 trials in total) of each condition.

Monetary amounts presented during the study ranged from $0 to $100 and were converted to real payouts using an exchange rate of 50 to 1. Participants learned in the instructions prior to the task that there would be an exchange rate but were not informed of the precise ratio until the end of the study. Participants were additionally compensated $5 (U.S.) for their time. All participants provided informed consent as approved by the research ethics board at the University of Toronto.

In Study 2, we sought to conceptually replicate Study 1 with a more natural measure of information search and more statistical power. We recruited a random sample of participants from the United States and Canada through Prolific Academic (final *N* = 204; gender: 111 men, 89 women, four nonbinary; age: *M* = 29.2 years, *SD* = 9.57, range = 18–78 years). Each participant was randomly assigned to play either the dictator game (*n* = 102) or ultimatum game (*n* = 102). In this experiment, sample size was determined through bootstrapped power analyses (Strong & Alvarez, 2019) on pilot data collected with a smaller sample (*N* = 40). These power analyses revealed that a sample size of 200 (100 for each condition) would yield a simultaneous power of .775 for all of our key analyses. To allow for more naturalistic information search, instead of having participants click in predefined AOIs to reveal information about the respective attributes, we had participants in Study 2 reveal information about the attributes simply by hovering their mouse over the predefined AOIs. Thus, in this version, participants were allowed to freely control the duration of exposure for each information sample. Information samples were defined as hovering over a predefined AOI for 100 ms or more (Manor & Gordon, 2003). Any instance of information sampling lasting less than 100 ms was discarded in analysis. Our results were robust to alternative specifications of the sample threshold and replicated when we defined any instance of information sampling as hovering over a predefined AOI for 50 ms or more. Furthermore, to account for the reduction in motor requirements for hovering compared with clicking, we reduced the time limit in the high-time-pressure condition of Study 2 from 3 s to 1.5 s. Correspondingly, in the low-time-pressure condition, participants had 10 s to decide but were prohibited from responding within the first 1.5 s. All other features of Study 2 were identical to those of Study 1.

### Experimental stimuli

The monetary amounts in both Studies 1 and 2 consisted of 20 unique combinations of $Self and $Other that were repeated 10 times, five in each time-pressure condition. Each instance, however, was modified by some uniformly distributed random integer values, *U*(–2, 2), to each of the values. The resulting stimulus pairs were thus such that if $Self was greater than 50, $Other would be less than or equal to 50, and if $Other was greater than 50, $Self would be less than or equal to 50. The remaining 20 trials consisted of catch trials in which the proposals for $Self and $Other were equally greater than $50 in 10 trials and equally less than $50 in the other 10 trials (i.e., offers could be [75, 75] or [10, 10] but never [65, 85] or [35, 10]). The exact amount for these catch trials was determined using a random uniform distribution.

All task stimuli were defined in proportions relative to screen size in order to account for the potential differences in the size of participants’ devices. The AOIs were defined as rectangles on the left and right sides of the screen with a width of 27% of the screen width and a height of 42% of the screen height. The boundaries of the AOIs were horizontally separated from each other by a relative width of 15% and from the center by 7.5%. Both AOIs were vertically centered. Each AOI was highlighted by a border to indicate its position to participants for their mouse movements. Stimuli were presented and responses collected using *Inquisit Web* (Version 5.0.14.0).

### Time-pressure manipulation check

In Study 1, participants in the dictator game took on average 1,734 ms (*SD* = 270 ms) to respond under high time pressure and 3,584 ms (*SD* = 273 ms) under low time pressure, whereas participants in the ultimatum game took on average 1,768 ms (*SD* = 273 ms) to respond under high time pressure and 3,558 ms (*SD* = 217 ms) under low time pressure. In Study 2, participants in the dictator game took on average 1,028 ms (*SD* = 147 ms) to respond under high time pressure and 2,185 ms (*SD* = 355 ms) under low time pressure, whereas participants in the ultimatum game took on average 1,005 ms (*SD* = 181 ms) to respond under high time pressure and 2,209 ms (*SD* = 397 ms) under low time pressure.

Further analyses of log-transformed response times (logRTs) revealed that time pressure led to significantly faster responses in both the dictator game—Study 1: *b* = −0.749, *SE* = 0.003, *t*(21898) = −221.294, two-tailed *p* < .001, *r* = −.831, 95% confidence interval (CI) = [−.834, −.829]; Study 2: *b* = −0.752, *SE* = 0.003, *t*(44674) = −254.224, two-tailed *p* < .001, *r* = −.769, 95% CI = [−.771, −.767]—and ultimatum game—Study 1: *b* = −0.722, *SE* = 0.003, *t*(21898) = −213.287, two-tailed *p* < .001, *r* = −.822, 95% CI = [−.824, −.819]; Study 2: *b* = −0.793, *SE* = 0.003, *t*(44674) = −268.018, two-tailed *p* < .001, *r* = −.785, 95% CI = [−.787, −.783].

### Prosocial choices

We defined prosocial choices as trials in which the participant accepted a smaller amount of money for themselves or rejected a larger amount for themselves compared with the default, in order to help their partner receive a larger amount of money. Choices were defined as selfish otherwise. Catch trials were removed from analyses of prosociality because prosocial behavior in this context is undefined. Missed response trials (mean percentage of trials—high time pressure: Study 1 = 1.627%, Study 2 = 5.562%; low time pressure: Study 1 = 0.255%, Study 2 = 0.049%) were excluded from choice analyses.

### Information search

We defined first information samples as the first piece of information that participants sampled by clicking or hovering their mouse over the predefined AOI ($Self or $Other). In Study 2, first information sample durations were defined as the amount of time that participants’ mouse spent within the same AOI in that first information sample prior to movement outside the AOI.

### Exclusions

In Study 1, we excluded 86 participants out of a total of 186 recruits on the basis of preregistered criteria (two did not complete task; one revoked consent for data use; 17 failed the comprehension check; 22 provided the same response in > 90% of all trials; one failed to respond in time > 25% of trials in one time-pressure condition; 43 failed catch trials). In Study 2, we excluded 187 participants out of a total of 391 recruits on the basis of preregistered criteria (three duplicate submissions; 43 failed the comprehension check; 51 provided the same response in > 90% of all trials; 15 failed to respond in time > 25% of trials in one condition; 75 failed catch trials). Importantly, the greater occurrence of missed trials in the time-pressure condition did not explain any behavioral effects reported below, which replicated when imputing the likely response on missed high-time-pressure trials from the observed response on the closest corresponding trial in the low-time-pressure condition (defined by the trial with the minimum Euclidean distance from the missed trial in terms of $Self and $Other). We further excluded trials post hoc in Studies 1 and 2 in which choices were made by guessing, defined as a choice made prior to sampling any information (high time pressure: *M*_Study1_ = 1.463%; *M*_Study2_ = 16.493%; low time pressure: *M*_Study1_ = 0.0455%; *M*_Study2_ = 0.160%) in analyses of aggregate prosociality. Inclusion of guess trials changed the statistical significance of the analysis of aggregate prosociality in Study 2 but did not otherwise change conclusions. For full disclosure, we additionally report analyses for Studies 1 and 2 exactly as preregistered without this post hoc exclusion in the Results section.

### Statistics

We conducted general linear mixed-effects regressions in the R programming environment (Version 3.6.3; R Core Team, 2020) using the *lme4* package (Version 1.1-21; Bates et al., 2015; Kunzetsova et al., 2017) with degrees of freedom estimated using the Satterthwaite method and generalized Poisson mixed-effects regression in R using the *glmmTMB* package (Version 1.0.0; Brooks et al., 2017). Effect size using *r* values was calculated for mixed-effects linear models using the transformation of *t* statistics and mixed-effects logistic models using transformation of odds ratios (Borenstein et al., 2011; Edwards et al., 2008; Kashdan & Steger, 2014). Effect size using incidence-rate ratio (IRR) was calculated for mixed-effects quasi-Poisson regressions (Olivier et al., 2017). Model comparison was conducted using the Bayesian information criterion (BIC; Burnham & Anderson, 2004; Raftery, 1996).

## Results

### Manipulation check: time pressure reduces the total amount of information acquired before choice

Our theory rests on the assumption that information-seeking priorities, particularly under time pressure, are driven by constraints on information processing. We thus began by verifying that time pressure constrains information search through limiting the total number of information samples that participants could make. Results showed that, as expected, time pressure reduced the total number of information samples that participants made within a trial in both the dictator game (Study 1: simple effect *b*_time_ = −0.208, *SE* = 0.006, *z* = −33.377, preregistered one-tailed *p* < .001, IRR = 0.812, 95% CI = [0.802, 0.822]; Study 2: simple effect *b*_time_ = −0.462, *SE* = 0.006, *z* = −75.270, preregistered one-tailed *p* < .001, IRR = 0.630, 95% CI = [0.622, 0.638]) and ultimatum game (Study 1: simple effect *b*_time_ = −0.189, *SE* = 0.006, *z* = −30.162, preregistered one-tailed *p* < .001, IRR = 0.828, 95% CI = [0.818, 0.838]; Study 2: simple effect *b*_time_ = −0.455, *SE* = 0.006, *z* = −76.690, preregistered one-tailed *p* < .001, IRR = 0.635, 95% CI = [0.627, 0.642]; for model details, see Table S1). Similarly, time pressure also increased the occurrence of incomplete information search (i.e., choosing before sampling information about both self and other) in both the dictator game (Study 1: simple effect *b*_time_ = 3.423, *SE* = 0.150, *z* = 22.819, two-tailed *p* < .001, *r* = .686, 95% CI = [.653, .716]; Study 2: simple effect *b*_time_ = 4.616, *SE* = 0.085, *z* = 54.190, preregistered one-tailed *p* < .001, *r* = .786, 95% CI = [.775, .797]) and ultimatum game (Study 1: simple effect *b*_time_ = 3.679, *SE* = 0.242, *z* = 15.184, two-tailed *p* < .001, *r* = .712, 95% CI = [.662, .753]; Study 2: simple effect *b*_time_ = 4.382, *SE* = 0.083, *z* = 52.864, preregistered one-tailed *p* < .001, *r* = .770, 95% CI = [.758, .782]; for model details, see Table S1). These results confirmed our predictions that participants truncate their information search to cope with time constraints and sometimes expedite choices by choosing even when they have incomplete information about amounts of money for self or other.

### Prediction 1: people prioritize acquiring the most relevant information to cope with time constraints

Our theory predicts that people optimize the order of their search to prioritize the most relevant information, particularly under time and resource constraints, and it is this serial ordering, rather than automatic impulses, that is the primary driver of changes in social behavior under time pressure. We thus sought to test the first set of predictions falling out of our theory: that different social contexts lead to different patterns of information search, particularly under time pressure. Although we assumed that most people’s ultimate goal is to maximize their own earnings, we expected that information about self and other outcomes is differentially relevant to that goal in altruistic versus cooperative choice contexts and that this produces different information priorities. This leads to four specific, but related, predictions. First, people should overall tend to acquire information about their own outcomes first. Second, people should in general be more likely to first acquire information about the other’s outcomes in the ultimatum game compared with the dictator game (because one’s own outcomes depend on acceptance by the other in the ultimatum game). Third, time pressure should exacerbate this difference because it magnifies the costs of acquiring information. Finally, information-search priorities under time pressure should influence not only first information samples but also whether people make choices without acquiring all relevant information. In particular, our theory suggests that people might be more likely to engage in additional information samples to the unknown attribute in the ultimatum game (where both pieces of information are highly relevant to the final outcome) compared with the dictator game.

Across Studies 1 and 2, we found strong support for all four predictions. Whereas participants in general were biased toward looking at their own outcomes first (Study 1: *b*_0_ = −1.482, *SE* = 0.764, *z* = −1.941, two-tailed *p* = .052, *r* = −.378, 95% CI = [-.635, .004]; Study 2: *b*_0_ = −0.919, *SE* = 0.216, *z* = −4.257, two-tailed *p* < .001, *r* = −.246, 95% CI = [-0.347, -0.135]), we found that game context shaped first information biases, regardless of time pressure. Participants in the ultimatum game were nonsignificantly less self-biased compared with participants in the dictator game in Study 1, and significantly so in Study 2, which was better powered to detect effects (Study 1: *b*_game_ = 0.968, *SE* = 1.467, *z* = 0.660, two-tailed *p* = .509, *r* = .258, 95% CI = [-.465, .727]; Study 2: *b*_game_ = 0.917, *SE* = 0.417, *z* = 2.202, preregistered one-tailed *p* = .014, *r* = .245, 95% CI = [.028, .431]).

**Fig. 2.**
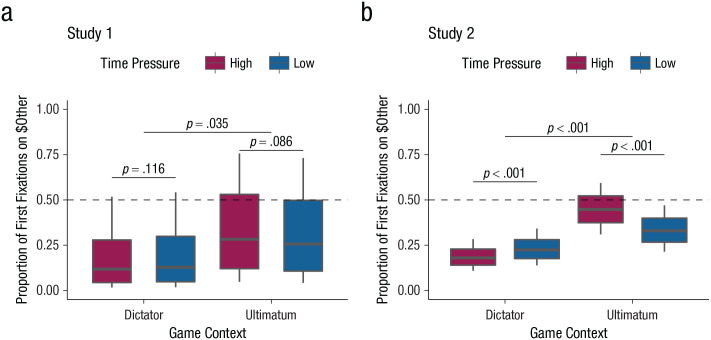
Information-search priorities as a function of game context and time pressure in (a) Study 1 and (b) Study 2. Box plots represent the proportion of trials in which participants first fixated on $Other. The central line indicates the mean, the upper and lower boundaries indicate one within-subjects standard error above and below the mean, and the whiskers indicate the within-subjects 95% confidence interval. All statistical tests were preregistered one-tailed tests.

More importantly, and as predicted, time pressure exacerbated the influence of game context on participants’ first information samples (see Fig. 2; Study 1: interaction *b*_time:game_ = 0.228, *SE* = 0.126, *z* = 1.816, preregistered one-tailed *p* = .035, *r* = .063, 95% CI = [-.005, .130]; Study 2: interaction *b*_time:game_ = 0.771, *SE* = 0.064, *z* = 11.986, preregistered one-tailed *p* < .001, *r* = .208, 95% CI = [.175, .240]; for model details, see Table S2; for post hoc analyses controlling for experimental block, see Note S2 in the Supplemental Material). Specifically, in the dictator game, participants prioritized searching for their own outcomes first more under time pressure (Study 1: simple effect *b*_time_ = −0.096, *SE* = 0.081, *z* = −1.194, preregistered one-tailed *p* = .116, *r* = −.027, 95% CI = [-.070, .017]; Study 2: simple effect *b*_time_ = −0.274, *SE* = 0.043, *z* = −6.328, preregistered one-tailed *p* < .001, *r* = −.075, 95% CI = [-.098, -.052]), whereas in the ultimatum game, participants prioritized searching for their partners’ outcomes first more under time pressure (Study 1: simple effect *b*_time_ = 0.132, *SE* = 0.096, *z* = 1.368, preregistered one-tailed *p* = .086, *r* = .036, 95% CI = [-.016, .088]; Study 2: simple effect *b*_time_ = 0.497, *SE* = 0.048, *z* = 10.433, preregistered one-tailed *p* < .001, *r* = .136, 95% CI = [.111, .161]).

However, consistent with our prediction that people might overall care more about their own outcomes, we note here that time pressure did not result in an overwhelming attentional prioritization of others’ outcomes even in the ultimatum game (Study 1: 24/50 participants still looked at their own outcomes first in > 50% of trials; Study 2: 59/102 participants). Moreover, we also found that people adopted additional search strategies in subsequent information samples to complement context-sensitive prioritization of first information samples when coping with time pressure during choice. Information priorities clearly shaped these complementary strategies to optimize information search (for full details, see Notes S1 and S2 in the Supplemental Material). More specifically, and as we predicted, participants in the ultimatum game appeared more motivated to acquire both pieces of information even under time pressure: They made more subsequent information samples in Study 1 (Study 1: interaction *b*_time:game_ = 0.019, *SE* = 0.009, *z* = 2.147, preregistered one-tailed *p* = .016, IRR = 1.019, 95% CI = [1.002, 1.037]; Study 2: interaction *b*_time:game_ = 0.008, *SE* = 0.008, *z* = 0.885, preregistered one-tailed *p* = .188, IRR = 1.008, 95% CI = [0.991, 1.024]) and were more likely to fixate on both pieces of information in Study 2 (Study 1: interaction *b*_time:game_ = 0.256, *SE* = 0.284, *z* = 0.900, preregistered one-tailed *p* = 1, two-tailed *p* = .368, *r* = .070, 95% CI = [-.083, .219]; Study 2: interaction *b*_time:game_ = −0.234, *SE* = 0.118, *z* = −1.979, preregistered one-tailed *p* = .024, *r* = −.064, 95% CI = [-.127, -.001]; for model details, see Table S1). We will return to these points in the Discussion section.

### Prediction 2: information priorities drive prosociality, particularly under time pressure

**Fig. 3.**
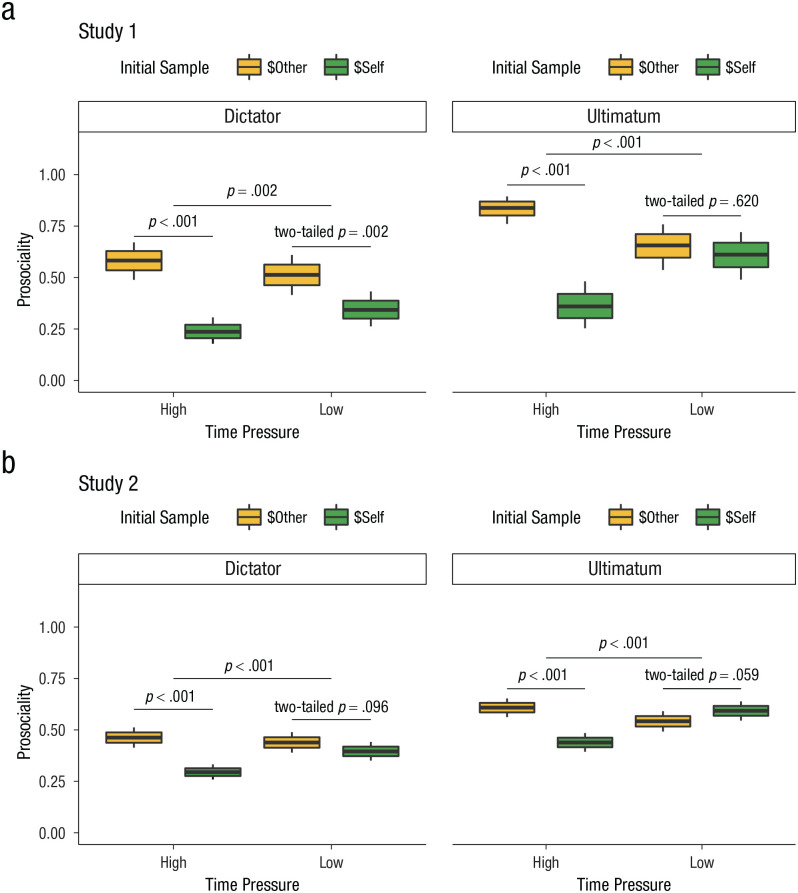
Trial-level effects of first information sample on prosociality as a function of game context and time pressure in (a) Study 1 and (b) Study 2. Box plots represent the probability of a prosocial choice. The central line indicates the mean, the upper and lower boundaries indicate one within-subjects standard error above and below the mean, and the whiskers indicate the within-subjects 95% confidence interval. All statistical tests were preregistered one-tailed tests unless otherwise indicated. Two-tailed tests conducted were post hoc comparisons.

Having shown support for our hypotheses that the context of social interactions (ultimatum game vs. dictator game) shapes adaptations in information-search processes under time pressure, we next examined our predictions that information priorities drive prosocial choices, specifically under time pressure across both contexts. In particular, we expected that because time pressure reduces information search, which information is acquired first should have a much larger effect on prosociality under time pressure than it does under free response. This should be true regardless of game context. As expected, across both studies, we found that time pressure interacted with first information samples to predict prosocial choices on each trial in both the dictator game (see Fig. 3; Study 1: simple interaction *b*_info1:time_ = 0.804, *SE* = 0.281, *z* = 2.861, preregistered one-tailed *p* = .002, *r* = .216, 95% CI = [.070, .350]; Study 2: simple interaction *b*_info1:time_ = 0.546, *SE* = 0.132, *z* = 4.144, preregistered one-tailed *p* < .001, *r* = .149, 95% CI = [.079, .216]) and the ultimatum game (see Fig. 3; Study 1: simple interaction *b*_info1:time_ = 2.030, *SE* = 0.510, *z* = 3.979, preregistered one-tailed *p* < .001, *r* = .488, 95% CI = [.273, .641]; Study 2: simple interaction *b*_info1:time_ = 0.895, *SE* = 0.138, *z* = 6.466, preregistered one-tailed *p* < .001, *r* = .239, 95% CI = [.169, .306]). In both games, participants were more likely to choose the prosocial option when they first looked at their partners’ outcomes compared with their own, specifically in the high-time-pressure condition (see Fig. 3; dictator game: simple effect of first information sample under high time pressure in Study 1: *b*_info1_ = 1.507, *SE* = 0.188, *z* = 7.995, preregistered one-tailed *p* < .001, *r* = .384, 95% CI = [.299, .459]; Study 2: *b*_info1_ = 0.724, *SE* = 0.084, *z* = 8.585, preregistered one-tailed *p* < .001, *r* = .196, 95% CI = [.152, .238]; ultimatum game: simple effect of first information sample under high time pressure in Study 1: *b*_info1_ = 2.223, *SE* = 0.384, *z* = 5.789, preregistered one-tailed *p* < .001, *r* = .523, 95% CI = [.376, .634]; Study 2: *b*_info1_ = 0.687, *SE* = 0.088, *z* = 7.828, preregistered one-tailed *p* < .001, *r* = .186, 95% CI = [.141, .230]), but less in the low-time-pressure condition (see Fig. 3; dictator game: simple effect of first information sample under low time pressure in Study 1: *b*_info1_ = 0.703, *SE* = 0.228, *z* = 3.079, two-tailed *p* = .002, *r* = .190, 95% CI = [.070, .302]; Study 2: *b*_info1_ = 0.178, *SE* = 0.107, *z* = 1.665, two-tailed *p* = .096, *r* = .049, 95% CI = [-.009, .106]; ultimatum game: simple effect of first information sample under low time pressure in Study 1: *b*_info1_ = 0.193, *SE* = 0.385, *z* = 0.501, two-tailed *p* = .617, *r* = .053, 95% CI = [-.153, .253]; Study 2: *b*_info1_ = −0.208, *SE* = 0.110, *z* = −1.886, two-tailed *p* = .059, *r* = −.057, 95% CI = [-.116, .002]).

Several analyses also suggested that, after we accounted for differences in information priorities, game context did not independently influence changes in prosociality under time pressure. First, although the three-way interaction between first information sample, time pressure, and game context was significant in Study 1 and marginally so in Study 2 (Study 1: three-way interaction *b*_info1:time:game_ = 1.226, *SE* = 0.599, *z* = 2.049, two-tailed *p* = .040, *r* = .320, 95% CI = [.015, .552]; Study 2: three-way interaction *b*_info1:time:game_ = 0.349, *SE* = 0.191, *z* = 1.831, preregistered one-tailed *p* = 1, two-tailed *p* = .067, *r* = .096, 95% CI = [-.007, .195]; for model details, see Table S3; for post hoc analyses controlling for block and trial-level variables, see Note S2), post hoc model comparison with models that included varied interactions between game context and other terms identified the model that included game context only as a main effect as the most parsimonious (Model C—Study 1: BIC_min_ = 23,420.46, Study 2: BIC_min_ = 45,444.26; for model details, see Table S3). Inclusion of the two-way interaction term between game context and time pressure strongly decreased the parsimony of the model without explaining significantly more variance (Model B—Study 1: ΔBIC = 7.37, Study 2: ΔBIC = 10.43). Inclusion of the three-way interaction between game context, first information sample, and time pressure and all its associated two-way interactions also further decreased the parsimony of the model without explaining significantly more variance (Model A—Study 1: ΔBIC = 25.57, Study 2: ΔBIC = 30.79). Thus, we found evidence that game context influences what people look at first, and what people look at first influences the likelihood of a prosocial choice, particularly under time pressure.

### Prediction 3: game context shapes divergent effects of time pressure on aggregate prosociality

Having shown that the game context shapes time pressure’s effects on information search and its subsequent effects on trial-level choice behavior, we next examined its effects on changes in aggregate prosociality under time pressure. This represents our core theoretical test for when and why time pressure might produce different effects on prosocial behavior in different game contexts. Critically, on the basis of an analysis of information acquisition patterns, we made several predictions about the effects of time pressure on overall prosociality in the dictator game versus ultimatum game. First, because people are overall self-oriented in information acquisition during the dictator game and become more so under time pressure, we predicted that time pressure should reduce the frequency of prosocial choices in this context. Second, because people are overall self-oriented in information acquisition during the ultimatum game but become less so under time pressure in our studies, we predicted that time pressure might have more equivocal effects, resulting either in decreased selfishness overall or in increased selfishness but to a much lesser degree in the ultimatum game compared with the dictator game.

**Fig. 4.**
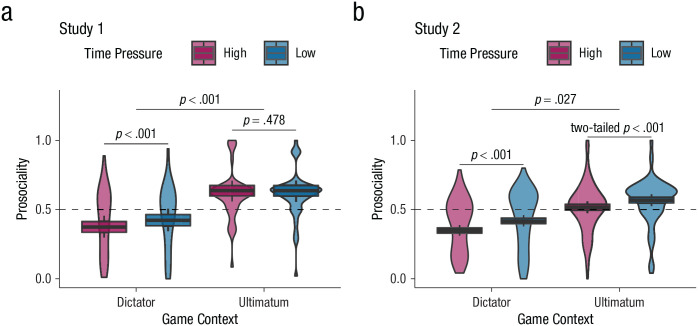
Group prosociality as a function of game context and time pressure in (a) Study 1 and (b) Study 2. Box plots represent the proportion of prosocial choices. The central line indicates the mean, the upper and lower boundaries indicate one within-subjects standard error above and below the mean, and the whiskers indicate the within-subjects 95% confidence interval. Violin plots illustrate the distribution of participants’ average generosity in each condition. All statistical tests were preregistered one-tailed tests unless otherwise indicated.

These predictions were strongly confirmed. Across both Study 1 and Study 2, we observed a significant two-way interaction between time pressure and game context in logistic mixed-effects regression predicting prosocial choices (see Fig. 4; Study 1: interaction *b*_time:game_ = 0.205, *SE* = 0.063, *z* = 3.235, preregistered one-tailed *p* < .001, *r* = .056, 95% CI = [.022, .090]; Study 2: interaction *b*_time:game_ = 0.090, *SE* = 0.047, *z* = 1.926, preregistered one-tailed *p* = .027, *r* = .025, 95% CI = [.000, .050]; for model details, see Table 1). Specifically, consistent with our predictions and past work (Teoh et al., 2020), we found that time pressure decreased prosociality in the dictator game (Study 1: simple effects *b*_time_ = −0.203, *SE* = 0.046, *z* = −4.431, preregistered one-tailed *p* < .001, *r* = −.056, 95% CI = [-.080, -.031]; Study 2: simple effects *b*_time_ = −0.296, *SE* = 0.033, *z* = −8.908, preregistered one-tailed *p* < .001, *r* = −.081, 95% CI = [-.099, -.064]). However, as predicted, we found that time pressure had inconsistent effects on prosociality in the ultimatum game, slightly but insignificantly increasing it in Study 1 and decreasing it in Study 2, although to a lesser extent than in the dictator game (Study 1: simple effects *b*_time_ = 0.002, *SE* = 0.044, *z* = 0.054, preregistered one-tailed *p* = .478, *r* = .001, 95% CI = [-.023, .024]; Study 2: simple effects *b*_time_ = −0.206, *SE* = 0.033, *z* = −6.309, preregistered one-tailed *p* = 1, two-tailed *p* < .001, *r* = −.057, 95% CI = [-.074, -.039]). As noted above, these equivocal findings are consistent with our model and replicate some previous findings in the literature (Bouwmeester et al., 2017). Post hoc analysis of aggregate prosociality further showed that for the subset of participants who consistently prioritized others’ outcomes over their own in the ultimatum game (i.e., fixated on $Other > 65% of trials; Study 1 *n* = 24, Study 2 *n* = 37), behavior did become significantly more prosocial under time pressure (*b*_time_ = 0.089, *SE* = 0.043, *z* = 2.082, two-tailed *p* = .037, *r* = .025, 95% CI = [.001, .048]) in both studies, as predicted (interaction *b*_time:study_ = −0.052, *SE* = 0.086, *z* = −0.603, two-tailed *p* = .546, *r* = −.014, 95% CI = [-.060, .032]).

**Table 1.** Effects of Game Context and Time Pressure on Aggregate Prosociality in Studies 1 and 2

Parameter	Study 1		Study 2	
	Excluding guesses	Including guesses	Excluding guesses	Including guesses
Main effects				
Intercept	0.076 [−0.157, 0.310]	0.077 [−0.156, 0.310]	−0.152* [−0.271, −0.032]	−0.113* [−0.224, −0.003]
Time pressure	−0.100** [−0.162, −0.038]	−0.094** [−0.156, −0.032]	−0.251*** [−0.297, −0.206]	−0.187*** [−0.229, −0.145]
Game context	0.976*** [0.512, 1.440]	0.971*** [0.506, 1.436]	0.651*** [0.412, 0.890]	0.611*** [0.392, 0.831]
Time Pressure × Game Context	0.205** [0.081, 0.329]^a^	0.190** [0.066, 0.313]^a^	0.090^†^ [−0.002, 0.181]^b^	0.032 [−0.052, 0.116]^b^
Simple effects				
Time pressure (dictator game)	−0.203*** [−0.292, −0.113]^c^	−0.189*** [−0.277, −0.100]^c^	−0.296*** [−0.361, −0.231]^d^	−0.203*** [−0.264, −0.143]^d^
Time pressure (ultimatum game)	0.002 [−0.084, 0.089]^e^	0.001 [−0.085, 0.087]^e^	−0.206*** [−0.270, −0.142]^f^	−0.171*** [−0.230, −0.113]^f^

Note: The table shows unstandardized coefficients from a mixed-effects logistic regression on prosociality (selfish = 0, prosocial = 1). Game context (ultimatum = 0.5, dictator = −0.5) and time pressure (high = 0.5, low = −0.5) were effects coded. Simple effects indicate the effect of the target variable at the level of other variables specified in parentheses. Participants were treated as a random effect with varying intercepts. Values in parentheses are 95% confidence intervals.

a Study 1 preregistered Hypothesis 1c: one-tailed *p* < .001 (excluding guess), one-tailed *p* = .001 (including guess).

b Study 2 preregistered Hypothesis 1c: one-tailed *p* = .027 (excluding guess), one-tailed *p* = .228 (including guess).

c Study 1 preregistered Hypothesis 1a: one-tailed *p* < .001 (excluding guess), one-tailed *p* < .001 (including guess).

d Study 2 preregistered Hypothesis 1a: one-tailed *p* < .001 (excluding guess), one-tailed *p* < .001 (including guess).

e Study 1 preregistered Hypothesis 1b: one-tailed *p* = .478 (excluding guess), one-tailed *p* = .490 (including guess).

f Study 2 preregistered Hypothesis 1b: one-tailed *p* = 1 (excluding guess), one-tailed *p* = 1 (including guess).

† Preregistered: one-tailed *p* = .027, two-tailed *p* = .054.

* *p* < .05. **p < .01. ***p < .001 (two-tailed).

## Discussion

Researchers have long theorized that time pressure reveals the operation of automatic intuitions and have thus applied this manipulation to infer the automatic or controlled nature of prosocial choice. Yet these efforts have yielded conflicting conclusions. To explain these contradictions, we demonstrate that time pressure might have different effects on social behavior in different contexts, not because it reveals the influence of intuitive social preferences but because it induces context-sensitive changes in information prioritization. Specifically, we found that time pressure drove people to prioritize gathering information about their own outcomes over others’ in altruistic contexts such as the dictator game, when one’s own outcomes conflict with those of others. In contrast, time pressure increased the extent to which people prioritized gathering information about others’ outcomes in cooperative contexts such as the ultimatum game, where self-outcomes partially depend on others’ satisfaction with the proposal. Because time pressure also forces people to more frequently choose without acquiring both pieces of information (i.e., search truncation), these biased information priorities disproportionately influenced prosocial decisions under time constraints. Although we did not independently test the causal effects of these information-search processes on choice behavior, we interpret our findings causally in light of the extensive work showing attentions’ independent causal role in choice behavior, including in paradigms similar to the one employed in our studies (Fiedler & Glöckner, 2012; Ghaffari & Fiedler, 2018; Krajbich et al., 2010; Smith & Krajbich, 2019; Teoh et al., 2020; Weilbächer et al., 2021).

Our findings further corroborate and extend existing work suggesting that altruism and cooperation constitute distinct prosocial contexts that may require consideration of different factors and recruit different psychological processes (Harbaugh & Krause, 2000; Ruff et al., 2013; Sally & Hill, 2006; Yamagishi et al., 2016, 2017). They also suggest that time pressure’s divergent effects across contexts might derive more from the ways in which people adapt their information-search strategy to specific social contexts, in order to cope with processing constraints (Callaway et al., 2021). This framework has the potential to not only make sense of existing patterns in the literature but also predict whether time pressure will increase or decrease prosociality in new contexts.

Specifically, our attention-based framework explains why, contrary to some previous work, we found no evidence that time pressure increases overall prosociality, even in ultimatum games where cooperative prosociality increases maximal joint interest (Bouwmeester et al., 2017; Rand, 2016). Firstly, in our model, changes in prosociality under time pressure result from the disproportionate influence of the first-fixated information due to search truncation. Although we did find that individuals became more likely to look first at their partners’ outcomes in ultimatum games compared with dictator games under time pressure, they still on average showed selfish looking biases in both contexts. Our model predicts increases in overall prosociality under time pressure only if participants prioritize searching for their partners’ outcomes over their own. Thus, in the current studies, we would not expect a group-level increase in prosociality in the ultimatum game—only a more moderate decrease compared with the dictator game. This is exactly what we observed.

How then can we explain increases in prosociality in other studies? We speculate that these increases might derive from contextual differences across studies that emphasize other individuals’ outcomes. In our study, the strategic benefits of cooperating in the ultimatum game did not motivate uniform prioritization of other people’s outcomes over one’s own. But some contexts may provide more salient reasons for attending to other people’s outcome. For instance, people may start out attending to others’ outcomes but learn over time that they can devote more attention to their own in order to maximize profits. In studies measuring only a few choices, this would produce a more consistent bias to attend first to other individuals’ outcomes, especially under time pressure, resulting in increased prosociality. Future work should investigate how varying the salience and strategic advantage of cooperative prosociality influences information search and subsequent choices under time pressure. This may explain why some studies have found that experience with social games mitigates time pressure’s effects (Rand et al., 2014).

Importantly, our work highlights how resource-rational meta-level choices about when to truncate information search influence time pressure’s effects. Here, we found evidence that time pressure truncated search to a lesser extent in the ultimatum game compared with the dictator game, likely because information about self- and other outcomes had more equivalent relevance to final payoffs in the ultimatum game. Thus, even participants whose first information samples were biased toward their partners’ outcomes were more likely to acquire all information prior to making their choices in the ultimatum game, thereby reducing the disproportionate influence of first information samples under time pressure. We speculate that this, too, might explain the more inconsistent effects of time pressure on cooperative prosociality in both our work and the broader literature (Bouwmeester et al., 2017; Rand, 2016) compared with pure altruism (Chen & Krajbich, 2018; Teoh et al., 2020).

One potential limitation of our work here is that information search in our paradigm was constrained and measured using mouse movements, which incur greater temporal and metabolic costs in comparison with more naturalistic eye movements. However, we believe that our findings are not specific to information search using mouse movements. Notably, even when all information is simultaneously available, extensive work strongly suggests that in-depth information processing is highly constrained by sequential foveation, although some parallel processing in extrafoveal vision may occur to guide subsequent eye movements (Cornelissen et al., 2005; Geringswald et al., 2012; Ludwig et al., 2014; Rayner & Bertera, 1979). Existing work also demonstrates that mouse-contingent information search is highly consistent with lab-based eye-tracking in visual search paradigms (Anwyl-Irvine et al., 2021). Additionally, previous work has also reported similar effects of time pressure on search truncation and information prioritization during prosocial decision-making in eye-tracking experiments in which all information is simultaneously presented (Teoh et al., 2020). Thus, we think that our results likely extend beyond the limits of the paradigm used here, although future work will be needed to confirm this.

These findings add to the growing literature cautioning against the assumption that time pressure can be used in a straightforward way to arbitrate between automatic and reflective processes (Roberts et al., 2022; Teoh et al., 2020). However, our results do not preclude the possibility of automatic and deliberate processing during social decision-making. Indeed, some studies show that previously rewarded locations automatically capture attention regardless of goal context (Anderson et al., 2011). If information about one’s own outcomes is more rewarding than information about others’ outcomes, and the location of this information is consistent, automatic capture may bias early information search. Future work measuring information search in both ultimatum and dictator games will be needed to investigate how automatic attention-capture effects might compete with more goal-driven deployment of attention (Fiedler et al., 2013; Ghaffari & Fiedler, 2018).

Whereas we propose information prioritization and truncation as mechanisms underlying time pressure’s effects on prosocial choice, other manipulations such as cognitive load, ego depletion, and intuition induction have also been employed to delineate between automatic and deliberate prosociality (Rand, 2016). We emphasize here that our model would not necessarily make similar predictions across these distinct manipulations. For example, in contrast to time pressure, which leads to truncation of search processes, cognitive load may instead disrupt information retention (Camos & Portrat, 2015). This may then lead to less consistent choices due to information uncertainty (Olschewski et al., 2018). However, it is also possible that people may attempt to cope with cognitive load by alternating between taking shorter but more frequent samples of the task-relevant information and attending to internally represented load. Furthermore, the content of the load itself may also interact with and bias search (Soto & Humphreys, 2008). Thus, changes in prosocial choice behavior under cognitive load would depend on both the extent and content of the load manipulation. Future work will be needed to articulate and test the precise informational and representational mechanisms that are targeted by each of these specific manipulations and how they converge and/or diverge from time pressure.

Finally, we believe that our theories about the influence of contextually dependent, prioritized information search on choices apply more generally to other domains of decision-making in which researchers have speculated about the dynamics of automatic and controlled processing, including dietary choice (HajiHosseini & Hutcherson, 2021; Maier et al., 2020; Sullivan & Huettel, 2021), risky decision-making (Diederich & Trueblood, 2018; Yechiam & Hochman, 2013), and intertemporal choice (Zhao et al., 2019). They may also advance our understanding of social behavior more broadly, with applications for understanding how competitive versus cooperative workplace cultures shape conflict resolution (Tjosvold, 1998) or how public health messaging emphasizing individual versus collective responsibility shapes health behaviors (Cho & Salmon, 2007; Jordan et al., 2021). To do so, however, future work would also have to extend beyond highly controlled experiments utilizing online economic games because prosocial behavior in the real world is embedded in a complex ecology that is characterized by greater uncertainty and social dependency. Developing models that allow us to both explain and predict the dynamic deployment of attention across richer real-world contexts may yield a more ecological understanding of how processing constraints such as time pressure interact with the larger goal context to impact decision-making, leading to more effective interventions for improving choice behavior.

## Supplemental Material

sj-pdf-1-pss-10.1177_09567976221094782 – Supplemental material for The Games We Play: Prosocial Choices Under Time Pressure Reflect Context-Sensitive Information Priorities Supplemental material, sj-pdf-1-pss-10.1177_09567976221094782 for The Games We Play: Prosocial Choices Under Time Pressure Reflect Context-Sensitive Information Priorities by Yi Yang Teoh and Cendri A. Hutcherson in Psychological Science

## Transparency

*Action Editor:* Leah Somerville

*Editor:* Patricia J. Bauer

*Author Contributions*

Both authors developed the study concept and designed the study. Y. Y. Teoh collected and analyzed the data. Both authors prepared the manuscript and approved the final manuscript for submission.

**Open Practices:** All data, analysis code, and materials have been made publicly available via OSF and can be accessed at https://osf.io/ftxsc/. The design and analysis plans for the studies were preregistered (Study 1: https://osf.io/zfrhb/, Study 2: https://osf.io/zx7b8/). This article has received the badges for Open Data, Open Materials, and Preregistration. More information about the Open Practices badges can be found at http://www.psychologicalscience.org/publications/badges.

## Notes

1. Although theorists sometimes disagree on the precise boundaries of prosocial behavior (e.g., is strategically self-interested cooperation truly prosocial?), we here define prosocial behavior in terms of the extent to which the behavior itself benefits other individuals, rather than requiring that the underlying motive be purely other-regarding. Thus, offering more to another person in an ultimatum game, where they can reject unfair offers, would constitute prosociality for our purposes because it displays an awareness of and respect for another person’s likely mental states, feelings, and interest (for further discussion on the relation between prosocial motives and prosocial behavior, see Batson, 1987, 2011).
