# Supplementary material for: The Games We Play: Prosocial Choices Under Time Pressure Reflect Context-Sensitive Information Priorities
Source: Psychol Sci. 2022 Aug 22;33(9):1541–56. doi: 10.1177/09567976221094782 (PMC9630724; doi:10.1177/09567976221094782)
Supplement: sj-pdf-1-pss-10.1177_09567976221094782 – Supplemental material for The Games We Play: Prosocial Choices Under Time Pressure Reflect Context-Sensitive Information Priorities [file sj-pdf-1-pss-10.1177_09567976221094782.pdf]

Supplemental Materials for
“The games we play: Prosocial choices under time pressure reflect context−sensitive information priorities.”

Yi Yang Teoh & Cendri A. Hutcherson

Table S1: Effects of game context, time pressure and initial information sample on total information sample counts, incomplete information search and initial sampling durations in Study 1 & 2

Predictors	b [95% CI]				
	Total Fixation Count		Proportion Incomplete Search		Initial Sampling Duration
	Study 1	Study 2	Study 1	Study 2	Study 2
**Main Effects**					
Intercept	0.671*** [0.630, 0.712]	0.577*** [0.547, 0.607]	−8.339*** [−9.801,−6.876]	−2.490*** [−2.801,−2.179]	5.576*** [5.538, 5.615]
Time Pressure	−0.198*** [−0.207,−0.190]	−0.458*** [−0.467,−0.450]	−2.759** [−4.826,−0.692]	−0.527† [−1.145, 0.091]	−0.202*** [−0.209,−0.195]
Game Context	−0.015 [−0.096, 0.066]	0.034 [−0.025, 0.094]	3.551*** [3.271, 3.830]	4.499*** [4.382, 4.616]	−0.030 [−0.107, 0.046]
Initial Information Sample	0.036*** [0.020, 0.051]	0.062*** [0.050, 0.073]	0.650** [0.233, 1.068]	−0.108† [−0.221, 0.005]	−0.062*** [−0.072,−0.052]
Time Pressure × Game Context	0.019* [0.002, 0.036][c]	0.008 [−0.009, 0.024][p]	0.256 [−0.302, 0.813]	−0.234* [−0.466,−0.002][k]	−0.027*** [−0.041,−0.013][g]
Time Pressure × Initial Information Sample	−0.028** [−0.045,−0.011]	0.040*** [0.023, 0.057]	1.742*** [1.166, 2.317]	0.329** [0.116, 0.542]	−0.038*** [−0.053,−0.024]
Game Context × Initial Information Sample	−0.015 [−0.046, 0.017]	−0.045*** [−0.068,−0.022]	0.937* [0.102, 1.772]	0.964*** [0.739, 1.189]	0.139*** [0.119, 0.159]
Time Pressure × Game Context × Initial Information Sample	−0.178*** [−0.213,−0.144]	−0.126*** [−0.159,−0.092]	4.081*** [2.933, 5.228][e]	0.218 [−0.205, 0.643][m]	0.094*** [0.066, 0.123][h]
**Simple Effects**					
Time Pressure (Dictator Game)	−0.208*** [−0.220,−0.196][a]	−0.462*** [−0.474,−0.450][n]	3.423*** [3.129, 3.716]	4.616*** [4.449, 4.783][j]	−0.188*** [−0.198,−0.178][f]
Time Pressure (Ultimatum Game)	−0.189*** [−0.201,−0.176][b]	−0.455*** [−0.466,−0.443][o]	3.679*** [3.204, 4.154]	4.382*** [4.220, 4.544][j]	−0.215*** [−0.225,−0.206][f]
Time Pressure × Initial Information Sample (Dictator Game)	0.063*** [0.039, 0.086]	0.103*** [0.079, 0.127]	−0.299 [−0.851, 0.254][d]	0.220 [−0.071, 0.511][l]	−0.086*** [−0.106,−0.065]
Time Pressure × Initial Information Sample (Ultimatum Game)	−0.117*** [−0.142,−0.092]	−0.023† [−0.046, 0.000]	3.782*** [2.775, 4.789]	0.438** [0.126, 0.750]	0.009 [−0.011, 0.028]
Time Pressure × Game Context (Initial Information Sample: $Self)	0.108*** [0.087, 0.130]	0.070*** [0.050, 0.091]	−1.784*** [−2.538,−1.031]	−0.343** [−0.586,−0.101]	−0.074*** [−0.091,−0.057][h]
Time Pressure × Game Context (Initial Information Sample: $Other)	−0.070*** [−0.097,−0.043]	−0.055*** [−0.082,−0.029]	2.296*** [1.448, 3.144]	−0.125 [−0.499, 0.249]	0.020† [−0.002, 0.042][i]

Note: Mixed-effects generalized poisson regression on total information sample count. Mixed-effects logistic regression on incomplete information search (complete = 0; incomplete = 1). Mixed-effects linear regression on log transformed initial sampling duration (ms). Trials with no recorded information samples were discarded. Game context (Ultimatum = 0.5; Dictator = −0.5), time pressure (High = 0.5, Low = −0.5) and initial information sample ($Self = −0.5, $Other = 0.5) were effect coded. Simple effects indicate the effect of the target variable(s) at the level of other variables specified in parentheses. Participants were treated as a random effect with varying intercepts. †p < .10, *p < .05, **p < .01, ***p < .001 (two-tailed)

[a]Study 1 pre-registered hypothesis 2a: one-tailed p < .001
[b]Study 1 pre-registered hypothesis 2b one-tailed p < .001
[c]Study 1 pre-registered hypothesis 2c: one-tailed p = .016
[d]Study 1 pre-registered hypothesis 3b(i): one-tailed p = 1
[e]Study 1 pre-registered hypothesis 3b(ii): one-tailed p < .001
[f]Study 2 pre-registered hypothesis 2b(i): one-tailed p < .001
[g]Study 2 pre-registered hypothesis 2b(ii): one-tailed p < .001
[h]Study 2 pre-registered hypothesis 2b(iii): one-tailed p < .001
[i]Study 2 pre-registered hypothesis 2b(iii): one-tailed p = .038
[j]Study 2 pre-registered hypothesis 2c(i): one-tailed p < .001
[k]Study 2 pre-registered hypothesis 2c(ii): one-tailed p = .024
[l]Study 2 pre-registered hypothesis 2c(iii): one-tailed p = .069
[m]Study 2 pre-registered hypothesis 2c(iv): one-tailed p = .156
[n]Study 2 pre-registered hypothesis 3a: one-tailed p < .001
[o]Study 2 pre-registered hypothesis 3b: one-tailed p < .001
[p]Study 2 pre-registered hypothesis 3c: one-tailed p = .188

Table S2: Effects of game context and time pressure on initial information sample in Study 1 and 2.

Parameters	b [95% CI]	
	Study 1	Study 2
**Main Effects**		
Intercept	−1.482† [−2.979,0.014]	−0.919*** [−1.343,−0.496]
Time Pressure	0.018 [−0.106,0.141]	0.112*** [0.049, 0.175]
Game Context	0.968 [−1.907,3.843]	0.917* [0.101, 1.734]
Time Pressure × Game Context	0.228† [−0.018,0.475] [c]	0.771*** [0.645, 0.897] [f]
**Simple Effects**		
Time Pressure (Dictator Game)	−0.096 [−0.255,0.062][a]	−0.274*** [−0.358,−0.189][d]
Time Pressure (Ultimatum Game)	0.132 [−0.057,0.321][b]	0.497*** [0.404,0.590][e]

Note: Mixed−effects logistic regression regression on initial information sample ($Self = 0; $Other = 1). Trials with no recorded information samples were discarded. Game context (Ultimatum = 0.5; Dictator = −0.5) and time pressure (High = 0.5, Low = −0.5) were effect coded. Simple effects indicate the effect of the target variable(s) at the level of other variables specified in parentheses. Participants were treated as a random effect with varying intercepts.
†p < .10, *p < .05, **p < .01, ***p < .001 (two-tailed)
[a]Study 1 pre-registered hypothesis 3a(i): one-tailed p = .116
[b]Study 1 pre-registered hypothesis 3a(ii): one-tailed p = .086
[c]Study 1 pre-registered hypothesis 3a(iii): one-tailed p = .035
[d]Study 2 pre-registered hypothesis 2a(i): one-tailed p < .001
[e]Study 2 pre-registered hypothesis 2a(ii): one-tailed p < .001
[f]Study 2 pre-registered hypothesis 2a(iii): one-tailed p < .001

Table S3: Model selection of effects of game context, time pressure, and initial information sample on prosocial choice in Study 1

Parameters	b [95% CI]							
	Full		A		B		**C (Selected)**	
	Study 1	Study 2	Study 1	Study 2	Study 1	Study 2	**Study 1**	**Study 2**
**Main Effects**								
Intercept	0.091 [−0.130, 0.312]	−0.119* [−0.235,−0.004]	0.094 [−0.127,0.315]	−0.122* [−0.237,−0.006]	0.097 [−0.124,0.319]	−0.122* [−0.237,−0.006]	**0.098 [−0.123,0.319]**	**−0.122* [−0.237,−0.006]**
Time Pressure	−0.068* [−0.132,−0.003]	−0.175*** [−0.224,−0.127]	−0.064* [−0.128,0.000]	−0.174*** [−0.223,−0.126]	−0.064* [−0.128,0.000]	−0.176*** [−0.224,−0.128]	**−0.061† [−0.124,0.003]**	**−0.176*** [−0.224,−0.128]**
Initial Information Sample	1.157*** [0.833, 1.480]	0.345*** [0.246, 0.445]	1.164*** [0.888,1.439]	0.347*** [0.247, 0.446]	1.120*** [0.853,1.388]	0.347*** [0.248, 0.446]	**1.135*** [0.868,1.402]**	**0.347*** [0.248, 0.447]**
Game Context	0.902*** [0.461, 1.342]	0.609*** [0.378, 0.839]	0.905*** [0.465,1.346]	0.608*** [0.377, 0.838]	0.895*** [0.452,1.338]	0.606*** [0.375, 0.836]	**0.892*** [0.449,1.334]**	**0.604*** [0.374, 0.834]**
Remainder Fixation Bias	1.117*** [0.799, 1.435]	0.284*** [0.165, 0.403]	1.124*** [0.849,1.398]	0.287*** [0.168, 0.405]	1.089*** [0.820,1.358]	0.289*** [0.170, 0.408]	**1.109*** [0.840,1.377]**	**0.289*** [0.171, 0.408]**
Time Pressure × Initial Information Sample	1.417*** [0.824, 2.010]	0.720*** [0.533, 0.908]	1.079*** [0.580,1.577]	0.716*** [0.529, 0.903]	1.067*** [0.577,1.558]	0.718*** [0.531, 0.905]	**1.115*** [0.627,1.603]**	**0.721*** [0.535, 0.907]**
Time Pressure × Remainder Sampling Bias	1.321*** [0.712, 1.930]	0.447*** [0.227, 0.667]	0.981*** [0.464,1.498]	0.445*** [0.225, 0.665]	0.966*** [0.459,1.473]	0.451*** [0.232, 0.671]	**1.010*** [0.504, 1.515]**	**0.453*** [0.234, 0.672]**
Time Pressure × Game Context	0.105 [−0.024, 0.233]	0.002 [−0.095, 0.098]	0.103 [−0.025,0.232]	0.002 [−0.093, 0.098]	0.103 [−0.024,0.229]	0.012 [−0.080, 0.105]		
Initial Information Sample × Game Context	0.103 [−0.539, 0.745]	−0.212* [−0.409,−0.014]	0.192 [−0.109,0.494]	0.012 [−0.124, 0.149]				
Remainder Sampling Bias × Game Context	−0.085 [−0.717, 0.546]	−0.308* [−0.545,−0.071]						
Time Pressure × Initial Information Sample Bias × Game Context	1.226* [0.053, 2.400]	0.349† [−0.024, 0.723][f]	0.018 [−0.241,0.277]	−0.078 [−0.277, 0.120]				
Time Pressure × Remainder Fixation Bias × Game Context	1.270* [0.066, 2.475]	0.514* [0.077, 0.952]						
**Simple Effects**								
Time Pressure × Initial Information Sample (Dicator Game)	0.804** [0.253, 1.354][a]	0.546*** [0.288, 0.804][d]						
Time Pressure × Initial Information Sample (Ultimatum Game)	2.030*** [1.030,3.030][c]	0.895*** [0.624, 1.166][e]						
Initial Information Sample (High Time Pressure in Dictator Game)	1.507*** [1.138, 1.876][b]	0.724*** [0.559, 0.889]						
Initial Information Sample (High Time Pressure in Ultimatum Game)	2.223*** [1.470,2.976]	0.687*** [0.515, 0.859]						
**BIC**	23461.68	45484.45	23446.03	45475.05	23427.83	45454.69	**23420.46**	**45444.26**

Note: Mixed−effects logistic regression on prosocial choice on each trial (Selfish = 0; Prosocial = 1). Trials with no recorded information samples were discarded. Game context (Ultimatum = 0.5; Dictator = −0.5), time pressure (High = 0.5, Low = −0.5) and initial information sample ($Other = 0.5, $Self = −0.5) were effect−coded. We controlled for effects of remainder sampling biases, calculated by subtracting 0.5 from the proportion of samples of $Other over samples of $Self and $Other, not including the initial information sample (Only looked at $Self = −0.5; Only looked at $Other = 0.5). Simple effects indicate the effect of the target variable(s) at the level of other variables specified in parentheses. Participants were treated as a random effect with varying intercepts.†p < .10, *p < .05, **p < .01, ***p < .001 (two-tailed)

[a]Study 1 pre-registered hypothesis 4a: one-tailed p = .002 [full model]
[b]Study 1 pre-registered hypothesis 4a: one-tailed p < .001 [full model]
[c]Study 1 pre-registered hypothesis 4b: one-tailed p < .001 [full model]
[d]Study 2 pre-registered hypothesis 4a: one-tailed p < .001 [full model]
[e]Study 2 pre-registered hypothesis 4b: one-tailed p < .001 [full model]
[f]Study 2 pre-registered hypothesis 4c: one-tailed p = 1 [full model]

Note S1: Information priorities further shape subsequent complementary adaptations in information search.

While participants in our studies prioritized context-relevant information in initial information samples to cope with time pressure as we predicted, we also found that they adopted additional strategies to complement information prioritization during choice under time pressure. Specifically, participants in Study 2 tended to reduce the duration of their initial information sample under time pressure in both the Dictator and Ultimatum game (Dictator game: simple effect $b_{time} = -0.188$, SE = 0.005, $t(40915) = -37.151$, pre-registered one-tailed $p < .001$, $r = -0.181$, 95% CI [−0.190, −0.171]; Ultimatum game: simple effect $b_{time} = -0.215$, SE = 0.005, $t(40916) = -44.553$, pre-registered one-tailed $p < .001$, $r = -0.215$, 95% CI [−0.224, −0.206]), presumably to enable acquiring more information subsequently.

More critically, since information about participants' own outcomes and their partner's outcomes hold more equivalent value for choice in the Ultimatum game, we predicted that participants in the Ultimatum game would be more motivated to acquire both pieces of information. Confirming these predictions, we found that participants in the Ultimatum game tended to reduce their initial sampling durations more under time pressure compared to participants in the Dictator game (interaction $b_{time:game} = -0.027$, SE = 0.007, $t(40915) = -3.857$, pre-registered one-tailed $p < .001$, $r = -0.019$, 95% CI [−0.029, −0.009]; see Table S1 for model details). This reduction in initial sampling duration appeared to further serve the purpose of enabling participants to acquire *more diverse* information: participants in Ultimatum games tended to take more information samples (Study 1: interaction $b_{time:game} = 0.019$, SE = 0.009, $z = 2.147$, pre-registered one-tailed $p = .016$, IRR = 1.019, 95% CI [1.002, 1.037]; Study 2: interaction $b_{time:game} = 0.008$, SE = 0.008, $z = 0.885$, pre-registered one-tailed $p = .188$, IRR = 1.008, 95% CI [0.991, 1.024]) and were less likely to make choices with incomplete information under time pressure compared to participants in the Dictator game (Study 1: interaction $b_{time:game} = 0.256$, SE = 0.284, $z = 0.900$, pre-registered one-tailed $p = 1$ two-tailed $p = .368$, $r = 0.070$,

95% CI [−0.083, 0.219]; Study 2: interaction $b_{time:game}$ = −0.234, SE = 0.118, z = −1.979, pre-registered one-tailed p = .024, r = −0.064, 95% CI [−0.127, −0.001]; see Table S1 for model details).

Furthermore, we predicted that people's information priorities would additionally influence complementary strategies in subsequent information samples to dynamically cope with time pressure. Specifically, participants should not only seek to prioritize contextually relevant information it (i.e. $Self in Dictator games, and $Other in Ultimatum games) but should prioritize additional information gathering through subsequent information samples in a way that depends on the information acquired during the initial information sample. We found strong evidence for these predictions: what information participants acquired first on a trial interacted with the game context to moderate the effects of time pressure on subsequent aspects of information search, including initial sampling durations (Study 2: interaction $b_{time:game:info1}$ = 0.094, SE = 0.015, t(40960) = 6.476, pre-registered one-tailed p < .001, r = 0.032, 95% CI [0.022, 0.042]), total number of information samples (Study 1: interaction $b_{time:game:info1}$ = −0.178, SE = 0.018, z = −10.176, two-tailed p < .001, IRR = 0.837, 95% CI [0.809, 0.866]; Study 2: interaction $b_{time: game:info1}$ = −0.126, SE = 0.017, z = −7.365, two-tailed p < .001, IRR = 0.882, 95% CI [0.853, 0.912]) and the likelihood of incomplete search (Study1: $b_{time:game:info1}$ = 44.081, SE = 0.585, z = 6.971, pre-registered one-tailed p < .001, r = 0.747, 95% CI [0.629, 0.822]; Study 2: $b_{time:game:info1}$ = 0.218, SE = 0.217, z = 1.006, pre-registered one-tailed p = .157, r = 0.060, 95% CI [−0.057, 0.175]). In other words, whether people looked at the contextually higher or lower priority information first influenced how much additional information they acquired under time pressure,

Specifically, when participants acquired information about *their partner's outcomes* first in the Ultimatum game (i.e. where it is contextually more relevant, higher priority) compared to the Dictator game (i.e. where it is contextually less relevant, lower priority), time pressure reduced initial sampling durations less (Study 2: simple interaction $b_{time:game}$ = 0.020, SE = 0.011, t(40942) = 1.770, pre-registered one-tailed p = .038, r = 0.009, 95% CI [−0.001, 0.018]), reduced the number of subsequent information samples more (Study 1: simple $b_{time:game}$ = −0.070, SE = 0.014, z = −5.099, two-tailed p <

.001, IRR = 0.932, 95% CI [0.907, 0.958]; Study 2: simple $b_{time:game} = -0.055$, SE = 0.013, z = −4.109, two-tailed p < .001, IRR = 0.946, 95% CI [0.922, 0.972]) and increased incomplete search more (Study 1: simple interaction $b_{time:info1} = 2.296$, SE = 0.433, z = 5.309, two-tailed p < .001, r = 0.535, 95% CI [0.371, 0.655]; Study 2: simple interaction $b_{time:info1} = -0.125$, SE = 0.191, z = −0.657, two-tailed p = .511, r = −0.034, 95% CI [−0.136, 0.068]).

In contrast, when participants acquired information about *their own outcomes* first in the Ultimatum game (i.e. where it is contextually less relevant, lower priority) compared to the Dictator game (i.e. where it is contextually more relevant, higher priority), time pressure reduced the duration of their initial information sample more (Study 2: simple interaction $b_{time:game} = -0.074$, SE = 0.009, t(40934) = −8.575, pre-registered one-tailed p < .001, r = −0.042, 95% CI [−0.052, −0.033]), reduced the number of subsequent information samples less (Study 1: simple $b_{time:game} = 0.108$, SE = 0.011, z = 9.872, two-tailed p < .001, IRR = 1.114, 95% CI [1.090, 1.138]; Study 2: simple $b_{time:game} = 0.070$, SE = 0.010, z = 6.740, two-tailed p < .001, IRR = 1.073, 95% CI [1.051, 1.095]) and increased incomplete search less (Study 1: simple interaction $b_{time:info1} = -1.784$, SE = 0.384, z = −4.641, two-tailed p < .001, r = −0.441, 95% CI [−0.573, −0.273]; Study 2: simple interaction $b_{time:info1} = -0.343$, SE = 0.124, z = −2.772, two-tailed p = .006, r = −0.094, 95% CI [−0.159, −0.028]).

Together, these results suggest that people dynamically adapt their information search to cope with time pressure’s truncation of search processes, not only by prioritizing the most relevant information, but also by modulating the duration of each information sample and subsequent information search to ensure that they acquire the most context-relevant information before they make their choice. In the Ultimatum Game, this means they are more likely to require additional information before deciding if they look at their own outcomes first. In the Dictator Game, this means they are more likely to require additional information if they look at their partner’s outcomes first.

Note S2: Robustness of game context and time pressure's effects on information search and prosocial choice.

To demonstrate the robustness of the effects reported in the paper, we conducted additional post-hoc analyses controlling for experimental block number and trial level attributes (i.e. $Self and $Other), to determine whether these effects are driven solely by learning. While we explore some higher-order effects here that are consistent with our model, the data are severely underpowered to detect four-way interactions and do not constitute strong evidence for the presence of these effects. We thus report these results for completeness. Critically, these post-hoc analyses replicated our main findings that time pressure interacted with game context in predicting initial information samples, though this effect dropped to marginal significance in the less well-powered Study 1 (Study 1: $b_{time:game}$ = 0.193, SE = 0.127, z = 1.518, one-tailed p = .065, r = 0.053; Study 2: $b_{time:game}$ = 0.775, SE = 0.065, z = 12.010, one-tailed p < .001, r = 0.209; controlling for block). We also replicate our finding that these initial information samples predicted prosocial choices, especially under time pressure (Study 1: 2-way interaction $b_{info1:time}$ = 1.470, SE = 0.335, z = 4.393, one-tailed p < .001, r = 0.375; Study 2: 2-way interaction $b_{info1:time}$ = 0.583, SE = 0.104, z = 5.603, one-tailed p < .001, r = 0.159; when controlling for block, $Self & $Other).

We next examined if game context and time pressure differentially shaped information search and its effects on prosocial choice across blocks as participants learned about the task. Here, we considered two predictions our model makes about increased familiarity with the task, depending on time pressure's magnitude Specifically, as participants become experienced at the task, they gain a more accurate estimation of how much information they can sample while making a choice within the time limit. If they learn that time pressure severely limits search and they are consistently unable to complete their search, they may become more strategic in their information truncation and prioritization. In contrast, if they learn that time pressure is less severe and still allows complete search most of the time, they may truncate their search to a lesser extent and become less strategic in their

search priorities. Post-hoc analyses of information search including block as a moderator provides preliminary support for these predictions. While we had initially attempted to calibrate the time pressure manipulations in Study 1 & 2 to the motor costs of search (clicking vs hovering), our data suggests that time pressure in Study 1 is less severe than Study 2: participants in Study 1 complete search significantly more often than in Study 2 even under high time pressure (Study 1: $M_{complete}$ = 0.850 [SD = 0.312], Study 2: $M_{complete}$ = 0.457 [SD = 0.396]; $b_{time:study}$ = -0.739, SE = 0.171, z = -4.316, two-tailed p < .001, r = -0.200, 95% CI [-0.284, -0.110]).

Consistent with our post-hoc hypotheses on learning mentioned above, we found some evidence that this "incomplete search" effect was moderated by block in Study 2 when time pressure was severely limiting, but not Study 1 when time pressure was milder. In Study 2, participants learned over time to truncate more strategically by taking fewer total samples when they have acquired the context-relevant information, as evidenced by a significant 4-way interaction with block number in Study 2 but not Study 1 (Study 1: $b_{time:game:first:block}$ = −0.014, SE = 0.012, z = −1.198, two-tailed p = .231, IRR = 0.986, 95% CI [0.964, 1.009]; Study 2: $b_{time:game:first:block}$ = −0.024, SE = 0.011, z = −2.220, two-tailed p = .026, IRR = 0.976, 95% CI [0.955, 0.997]). In comparison to this finding examining total samples, we found no evidence that block moderated the effects of time pressure on incomplete search (Study 1: $b_{time:game:first:block}$ = 0.169, SE = 0.502, z = 0.336, two-tailed p = .737, r = 0.046, 95% CI [−0.219, 0.303]; Study 2: $b_{time:game:first:block}$ = 0.128, SE = 0.136, z = 0.944, two-tailed p = .345, r = 0.035, 95% CI [−0.038, 0.108]).

However, we did find that participants in Study 1 but not Study 2 became less likely to prioritize context-relevant information under time pressure in later blocks of the experiment, as measured by the information participants chose to sample first (Study 1: $b_{time:game:block}$ = − 0.439, SE = 0.090, z =-4.885, two-tailed p < .001, r = −0.120, 95% CI [−0.167, −0.072]; Study 2: $b_{time:game:block}$ = 0.069, SE = 0.044, z = 1.559, two-tailed p = .119, r = 0.019, 95% CI [−0.005, 0.043]). Specifically, in Study 1 when truncation was rarely required of participants, they became less likely to prioritize the

context relevant information over time, as might be expected if they learned that such prioritization was not as crucial.

Does the relationship between search patterns and choice change over time? Our results suggest not: we found no evidence that the block moderated the effect of time pressure, initial sampling biases or its two-way interactions on trial-level prosocial choice ($ps > .05$). In other words, choices remained conditional on information search patterns in a way that did not appear to change with learning over time.

Thus, taken together, we find some support for learning influencing aspects of the information sampling process across our experiments, consistent with our *post hoc* reasoning about the drivers of prioritized sampling. These effects also provide a potential explanation for the weaker effects observed in Study 1 compared to Study 2. We speculate here that since Study 1's time pressure manipulation was weaker than in Study 2 (3s vs 1.5s) and participants in Study 1 rarely chose without sampling both pieces of information, it is possible that participants in Study 1 became less motivated to prioritize context-relevant information under time pressure in the later blocks after learning across the blocks that they rarely needed to truncate search to choose within the time limit.

Finally, we sought to determine whether information search depends in important ways on the magnitude of the attributes that are sampled in each context. Specifically, we expected that time pressure may lead to truncation of search more so when participants first sample the context-relevant attribute ($Self in the dictator game and $Other in the ultimatum game) and the value of the attribute is large. However, our underpowered analyses failed to detect any significant moderations by trial-level attributes in either study for either $Self or $Other ($ps > .05$). Unsurprisingly, post-hoc analyses of trial-level prosociality revealed that participants were more likely to be prosocial when their own losses were smaller (Study 1: $b_{\$Self} = 2.586$, $SE = 0.051$, $z = 51.113$, two-tailed $p < .001$, $r = 0.580$, 95% CI [0.565, 0.595]; Study 2: $b_{\$Self} = 1.896$, $SE = 0.033$, $z = 57.014$, two-tailed $p < .001$, $r = 0.463$, 95% CI [0.451, 0.476]) and their partners' gains were larger (Study 1: $b_{\$Other} = 1.396$, $SE = 0.053$, $z = 26.152$,

two-tailed $p < .001$, $r = 0.359$, 95% CI [0.335, 0.382]; Study 2: $b_{\$Other} = 1.051$, $SE = 0.036$, $z = 28.913$, two-tailed $p < .001$, $r = 0.278$, 95% CI [0.261, 0.296]). We also found that time pressure reduced the effect of both these attributes on choice (Study 1: $b_{\$Self:time} = -0.551$, $SE = 0.096$, $z = -5.725$, two-tailed $p < .001$, $r = -0.150$, 95% CI [−0.200, −0.099]; $b_{\$Other:time} = -0.204$, $SE = 0.106$, $z = -1.929$, two-tailed $p = .054$, $r = -0.056$, 95% CI [−0.113, 0.001]; Study 2: $b_{\$Self:time} = -1.229$, $SE = 0.065$, $z =-18.861$, two-tailed $p < .001$, $r = -0.321$, 95% CI [−0.350, −0.291; $b_{\$Other:time} = -0.683$, $SE = 0.072$, $z = -9.440$, two-tailed $p < .001$, $r = -0.185$, 95% CI [−0.222, −0.148]), corroborating past work that time pressure results in a more noisy implementation of people's preferences. Also as expected, we found that participants in the ultimatum game tended to weigh their own losses less (Study 1: $b_{\$Self:game} = -1.197$, $SE = 0.101$, $z =-11.852$, two-tailed $p < .001$, $r = -0.313$, 95% CI [−0.359, −0.266]; Study 2: $b_{\$Self:game} = -0.671$, $SE = 0.066$, $z =-10.108$, two-tailed $p < .001$, $r = -0.182$, 95% CI [−0.216, −0.147]) and their partners' outcomes slightly more when deciding whether to make a prosocial choice (Study 1: $b_{\$Other:game} = 0.029$, $SE = 0.107$, $z = 0.275$, two-tailed $p = .783$, $r = 0.008$, 95% CI [−0.049, 0.066]; Study 2: $b_{\$Other:game} = 0.164$, $SE = 0.073$, $z = 2.263$, two-tailed $p = .024$, $r = 0.045$, 95% CI [0.006, 0.084]), consistent with the idea that this context leads to greater emphasis on others' outcomes.

These results demonstrate the robustness of our findings that information truncation and prioritization independently drive prosocial choice under time pressure. However, we also found some preliminary evidence in our post-hoc exploration of the data that people may be dynamically adapting these strategies over the course of the experiment as they learn more about the task structure. Future work will need to confirm these learning effects and investigate more precisely how the content of information samples influence the decision to extend or truncate search. Altogether, these results point towards context-sensitive information search and truncation as a critical mechanism that facilitates behavior under constraints (e.g., time pressure).
